# Evaluation of Superparamagnetic Silica Nanoparticles for Extraction of Triazines in Magnetic in-Tube Solid Phase Microextraction Coupled to Capillary Liquid Chromatography

**DOI:** 10.3390/nano4020242

**Published:** 2014-04-02

**Authors:** R. A. González-Fuenzalida, Y. Moliner-Martínez, Helena Prima-Garcia, Antonio Ribera, P. Campins-Falcó, Ramon J. Zaragozá

**Affiliations:** 1 Department of Analytical Chemistry. Faculty of Chemistry, University of Valencia, Dr. Moliner 50, Burjassot, Valencia E-46100, Spain; E-Mails: rodrigo.gonzalez@uv.es (R.A.G.-F.); yolanda.moliner@uv.es (Y.M.-M.); 2 Instituto de Ciencia Molecular (ICMol), University of Valencia, Catedrático José Beltrán 2, Paterna, Valencia E-46980, Spain; E-Mails: helena.prima@uv.es (H.P.-G.); antonio.ribera@uv.es (A.R.); 3 Department of Organic Chemistry, Faculty of Chemistry, University of Valencia, Dr. Moliner 50, E-46100 Burjassot, Valencia E-46100, Spain; E-Mail: ramon.j.zaragoza@uv.es

**Keywords:** magnetic nanoparticles, triazines, magnetic susceptibility, on-line solid phase microextraction, environmental samples

## Abstract

The use of magnetic nanomaterials for analytical applications has increased in the recent years. In particular, magnetic nanomaterials have shown great potential as adsorbent phase in several extraction procedures due to the significant advantages over the conventional methods. In the present work, the influence of magnetic forces over the extraction efficiency of triazines using superparamagnetic silica nanoparticles (NPs) in magnetic in tube solid phase microextraction (Magnetic-IT-SPME) coupled to CapLC has been evaluated. Atrazine, terbutylazine and simazine has been selected as target analytes. The superparamagnetic silica nanomaterial (SiO_2_-Fe_3_O_4_) deposited onto the surface of a capillary column gave rise to a magnetic extraction phase for IT-SPME that provided a enhancemment of the extraction efficiency for triazines. This improvement is based on two phenomena, the superparamegnetic behavior of Fe_3_O_4_ NPs and the diamagnetic repulsions that take place in a microfluidic device such a capillary column. A systematic study of analytes adsorption and desorption was conducted as function of the magnetic field and the relationship with triazines magnetic susceptibility. The positive influence of magnetism on the extraction procedure was demonstrated. The analytical characteristics of the optimized procedure were established and the method was applied to the determination of the target analytes in water samples with satisfactory results. When coupling Magnetic-IT-SPME with CapLC, improved adsorption efficiencies (60%–63%) were achieved compared with conventional adsorption materials (0.8%–3%).

## 1. Introduction

Magnetic nanomaterials are nowadays widely studied in many fields, such as medicine [1,2], materials science [3,4,5], environmental science [6,7] or chemistry [8,9,10]. Research based on these nanomaterials is a great deal bearing in mind the improvements that they can introduce in many applications of these fields. One of the most promising applications for magnetic nanomaterials is the use as sorbent materials, since they combine high adsorption capacity with the interesting magnetic properties of magnetic nanoparticles (NPs).

In recent years, one of the trends of analytical nanotechnology has been focused on developing magnetic nanomaterials as sorbent materials for sample pretreatment [11]. Particularly, these nanomaterials have been described for solid phase extraction (SPE), liquid-liquid microextraction (LLME) or solid phase microextraction (SPME) giving rise to the development of dispersive or magnetic SPE [7,12,13], dispersive LLME [14,15] and magnetic SPME [16,17] based procedures. These techniques take advantage of the large surface area of NPs and the interaction of magnetic nanomaterials with magnetic fields, and the advantages of its use have been demonstrated to improve conventional extraction procedures for several compounds in environmental, biological and clinical analysis.

While in the abovementioned approaches, the magnetic nanomaterials have been exploited in off-line modalities, there is a growing interest in developing on-line extraction procedures using magnetic nanomaterials, where the sample pretreatment step is coupled with the separation and/or detection technique. The main advantages of the on-line procedures rely on reduced analysis times and minimized sample handling, since samples can be directly processed. Furthermore, sensitivity and selectivity can be also improved. However, the challenge to be addressed in on-line devices is focused on the implementation of a magnetic field source.

In this context, several efforts have been made to develop on-line pretreatment techniques using magnetic nanomaterials, mainly in-capillary electrophoretic methods [18] and in-tube SPME (IT-SPME) [19,20] coupled to chromatographic techniques (Magnetic-IT-SPME). In the first approach, the magnetic field, supplied by permanent magnets nipped to the capillary, is used to generate a NPs coating on the surface of a capillary column, so the column efficiency is improved due to the large surface area of nanoparticles. The second approach is based on the functionalization of a capillary column with silica supported magnetic NPs. The capillary column, placed on a magnetic coil, is then used as the loop of the injection valve of a Capillary LC (CapLC). The main feature of this system is that the adsorption is not only governed by the surface area of the capillary column, but also the magnetic field plays an important role in the extraction efficiency and preconcentration, particularly for diamagnetic compounds. The adsorption mechanism is based on the partially adsorption of diamagnetic compounds on the silica supported magnetic NPs deposited capillary, combined with the influence of a magnetic field on superparamagnetic NPs. The magnetization of these NPs inside of the capillary generates regions with different magnetic field gradients, thus, diamagnetic analytes tend to be trapped in those regions where the magnetic field is minimal, yielding to improved adsorption efficiencies. Compared to typical off-line devices, Magnetic IT-SPME can be a powerful tool in the sample pretreatment step since it takes advantage of the high surface area of magnetic nanomaterials combined with the superparamagnetic behavior of magnetic NPs to develop more efficient on-line extraction techniques. This approach has been successfully proposed for several pharmaceutical compounds [19] and organophosphorous compounds [21] in the environmental field. Nevertheless, further investigations are still needed in order to advance in the knowledge of this technique and to extent potential applications in environmental studies and in other fields.

Herein, this work is focused on the study of the adsorption behavior of triazines on SiO_2_ supported Fe_3_O_4_ as sorbent material for magnetic-IT-SPME. A systematic study of triazines adsorption was conducted as function of the magnetic field and the relationship with triazines magnetic susceptibility. Finally, taking advantage of the enhanced adsorption properties, an analytical procedure have been developed and characterized for determining triazines in water samples.

## 2. Results and Discussion

### 2.1. Magnetic Characterization of the Capillary Columns and Target Analytes

**Figure 1:**
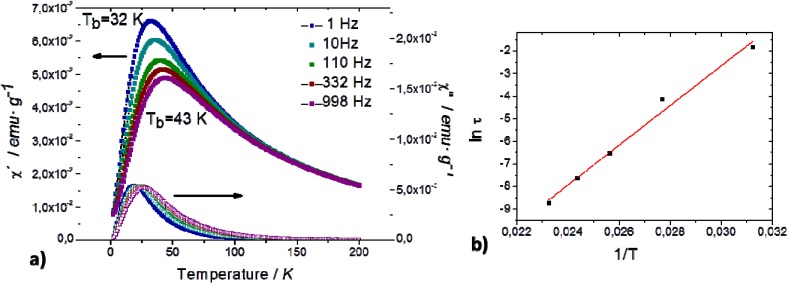
(**a**) Frequency dependence of AC susceptibility of the NPs *vs.* Temperature with amplitude of 17 Oe; (**b**) Arrhenius law fit for the nanoparticles.

Atrazine, simazine and terbutylazine are diamagnetic analytes, so susceptible to be extracted from aqueous medium with Magnetic-IT-SPME using SiO_2_ supported Fe_3_O_4_ deposited on the capillary column. Previous studies have reported that this sorbent nanomaterial improves the extraction efficiency of compounds such as acetylsalicylic acid, atenolol, acetaminophen, diclofenac, ibuprofen, chlorfenvinphos and chlorpyrifos [19,21]. It has been shown how this improvement is directly related with the influence of an external magnetic field on the SiO_2_ supported Fe_3_O_4_ nanomaterial deposited on the capillary column. Fe_3_O_4_ nanoparticles with an average size of 5 nm and in a percentage of 5 wt.% in the silica composite (data not shown), gave rise to the synthesis of magnetic capillary column [19]. The NPs inside of the capillary column are superparamagnetic and well isolated as can be deduced of the magnetic characterization of the NPs. In order to determine the type of interaction between NPs, dynamics of superspins have been studied by *AC* magnetic susceptibility measurements in frequency range of 1–1000 Hz. Figure 1a shows the frequency dependence of the *AC* susceptibility for the Fe_3_O_4_ nanoparticles. As one can see, by increasing of the applied frequency, the *T_B_* shifts to higher temperature. Relation between *T_B_* and relaxation time (inverse of applied frequency) for non-interacting nanoparticles is given by the Neel-Arrhenius law as: 
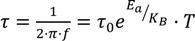
, where *E_a_* = *KV* is energy barrier (where *K* is magnetic anisotropy constant and *V* is the volume of nanoparticle), *K_B_* is Boltzmann constant, τ = 1/*f* and τ_0_ is about 10^−9^–10^−13^ s. Figure 1b shows the results of fitting the experimental data (real part) by the Arrhenius expression. The obtained values of τ_0_ and *E_a_* were 2.536·× 10^−13^ s and *E_a_*/*K_B_* = 878.22 ± 57.66 K, respectively. As mentioned above, the value of τ_0_ is about 10^−9^–10^−13^ s for noninteractiong nanoparticles. As we expected, no strong dipole-dipole interactions are between the magnetic nanoparticles. It has been already reported that this magnetic capillary columns are formed by well isolated and superparamagnetic NPs, thus are easily magnetized under the application of an external magnetic field.

By another hand, the nature of the analytes is the major factor affecting the extraction efficiency; pharmaceutical compounds showed a better enhancement of the adsorption capacity than organophosphours compounds when a magnetic field is applied. Therefore, in a first study, the magnetic susceptibilities of the different compounds were compared. Table 1 shows the energy level calculated for each compound.

**Table 1:** B3LYP/6-31G** total energies (*E*, au) *in vacuo* and in water.

Compound	In vacuo	In H_2_O
Acetylsalicilic acid	−648.709453	−648.720605
Acetaminophen	−515.494917	−515.507984
Diclofenac	−1665.736440	−1665.748421
Ibuprofen	−656.734267	−656.741794
Atenolol	−881.951314	−881.969818
Chlorpyrifos	−2671.570830	−2671.582537
Chlorfenvinphos	−2409.975077	−2409.986882
Simazine	−1007.990723	−1008.000696
Atrazine	−1047.309710	−1047.319747
Terbutylazina	−1086.624906	−1086.633924

Once we calculated the energy level, the magnetic susceptibilities for acetylsalicylic acid, atenolol, acetaminophen, diclofenac, ibuprofen, chlorfenvinphos and chlorpyrifos were evaluated, and these values were compared with the values for simazine, atrazine and terbutylazine. Table 2 shows the magnetic susceptibility values obtained for the studied compounds in vacuo and water. All the analytes are diamagnetic and as it was expected, there were not significant differences in the magnetic susceptibilities. Therefore, the differences on the adsorption capacity of the magnetic adsorbent toward each compound cannot be explained by the differences in the magnetic susceptibilities of these compounds, but must be crucially depending on the polarity of each compound and the chromatographic conditions, deeply influenced by the interaction of the external magnetic field with the magnetic adsorbent [19,22].

**Table 2:** Isotropic diamagnetic susceptibility (IDS), isotropic paramagnetic susceptibility (IPS) and isotropic total susceptibility (ITS) ^a^ in au.

Compound	In vacuo			In H_2_O		
	IDS	IPS	ITS	IDS	IPS	ITS
Acetylsalicilic acid	−375.1256	354.4428	−20.6827	−376.0739	355.3678	−20.7061
Acetaminophen	−361.7369	342.8417	−18.8952	−361.2246	342.2788	−18.9458
Diclofenac	−1073.6967	1037.0720	−36.6247	−1071.6524	1035.0105	−36.6419
Ibuprofen	−784.5031	754.6211	−29.8820	−784.0887	754.2092	−29.8795
Atenolol	−1895.5223	1858.5274	−36.9949	−1905.1243	1868.0617	−37.0626
Chlorpyrifos	−1070.6714	1033.9535	−36.7179	−1070.5282	1033.7223	−36.8059
Chlorfenvinphos	−1254.5588	1216.6838	−37.8751	−1257.4740	1219.5444	−37.9296
Simazine	−588.8682	563.8451	−25.0230	−591.2717	566.3096	−24.9621
Atrazine	−716.3337	688.7578	−27.5760	−717.3796	689.8408	−27.5388
Terbutylazine	−787.0130	757.0138	−29.9993	−786.0579	756.1178	−29.9401

^a^ ITS = IDS + IPS.

### 2.2. Adsorption of Triazines in the SiO_2_ Supported Fe_3_O_4_ Capillary Column

**Figure 2:**
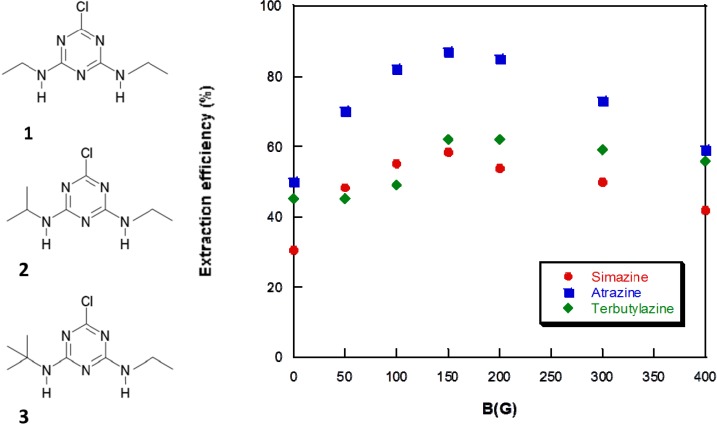
Variation of the extraction efficiency as function of the magnetic field for (**1**) simazine; (**2**) atrazine; and (**3**) terbutylazine. Injection 100 μL of a mixture of the target analytes (30 μg L^−1^). Mobile phase: methanol:water 85:15, flow 6 μL min^−1^. *B*_adsorption_ = *B*_desorption_ (reverse polarity).

Adsorption of simazine, atrazine and tertubylazine in the magnetic sorbent phase was studied following the procedure described in [19] with minimal modification. Typically, 100 μL of a mixture of triazines was passed through the capillary column in the load position (see Figure 5, Experimental Section) at variable magnetic fields (from 50 to 400 G). Once the sample is processed, the injection valve is rotated to the inject position (Figure 5, Experimental Section) at the same time that the polarity of the magnetic field is changed. Then, the adsorbed analytes are transferred from the magnetic capillary column to the chromatographic system for their separation and detection. Figure 2 shows the variation of the extraction efficiency as function of the magnetic field for the three target compounds. Note that the extraction efficiencies were calculated taking into account the slope of the calibration curve when standards at different concentration level were directly injected (2 μL) into the chromatographic system. As seen in Figure 2, the adsorption capacity increased as function of the magnetic field applied. Similar results have been reported [19,21]. These results indicated that the application of an external magnetic field induces the magnetization of Fe_3_O_4_ NPs inside of the capillary column, creating regions with different magnetic field gradients that depend on the intensity of the magnetic field. Under these conditions, diamagnetic analytes are inside of a paramagnetic medium, and they are submitted to repulsion forces, in such a way that analytes tend to be trapped in the minima of the magnetic field forces. This effect influenced the partitioning coefficients of the analytes in the mobile phase flow and the stationary phase with a maximum on the extraction efficiency at 150 G.

The enhancement on the adsorption led to an improvement of the extraction efficiency for simazine, atrazine and terbutylazine induced by the application of a magnetic field when the SiO_2_ supported Fe_3_O_4_ capillary column is used. In fact, control experiments showed that in absence of a magnetic field (B = 0 G), the percentage of extracted compound decreased till 30%, 50% and 45% for simazine, atrazine and terbutylazine, respectively. The mechanism by which analytes are entrapped into the sorbent material is based not only on the hydrophobic interactions between the analytes and the sorbent material, but also, on the influence of the magnetic field on the magnetic capillary column within the used configuration [19] (see Figure 5, Experimental Section).

**Figure 3:**
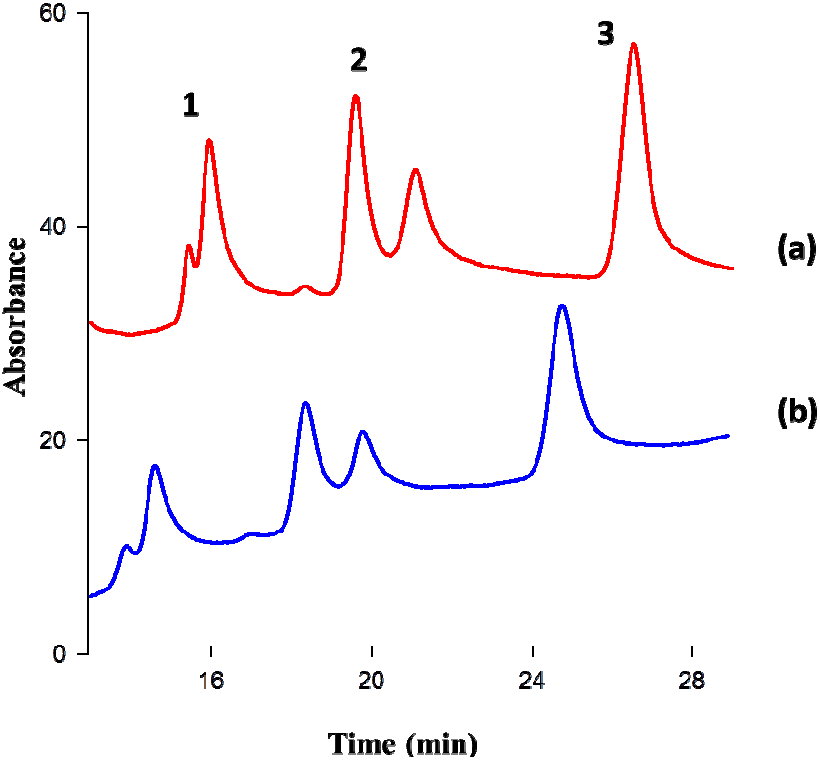
Chromatogram obtained with the magnetic capillary column in the Magnetic-IT-SPME device coupled with Cap-LC-DAD (230 nm); (**1**) simazine; (**2**) atrazine; and (**3**) terbutylazine. (**a**) Applying magnetic field *B*_adsoprtion_ = 150 G (*B*_desorption_ = 150 G, reverse polarity); (**b**) Without magnetic field (*B* = 0 G). Injection 100 μL of a mixture of the target analytes (30 μg L^−1^). Mobile phase: methanol:water 85:15, flow 6 μL min^−1^.

Regardless to the adsorption capacity of the SiO_2_ supported Fe_3_O_4_ material, it has been demonstrated that hydrophobic interactions between the analytes and the sorbent take place through the alkyl chain of CTAB that are structural units of the material [7]. In the case of Magnetic IT-SPME, a contribution of a difference force needs to be considered. Notice that the SiO_2_ matrix supported the Fe_3_O_4_ NPs and CTAB micelles. As it was demonstrated in [7], the material is formed by two types of micelles, CTAB micelles and Fe_3_O_4_-CTAB micelles and the main functions of SiO_2_ matrix were to support the NPs, but also to disperse and isolate Fe_3_O_4_ NPs. The injected compounds in the IT-SPME device are partially adsorbed on the surface of the capillary column. When a magnetic field is applied, the Fe_3_O_4_ NPs embedded on the silica matrix and deposited on the surface of the capillary column, are magnetized; this magnetization yield to the formation of different magnetic field gradients on the surface of the capillary column. Under these conditions, diamagnetic compounds are strongly affected, and they tend to be trapped in the minimal magnetic field regions, increasing the adsorption capacity. Once the analytes have been adsorbed, the desorption step was carried out by changing the polarity of the magnetic field. As it was previously demonstrated, that change is necessary to generate rapid changes in the magnetic strengths, and so the analytes can be detrapped for their subsequent separation and detection in the chromatographic system. Figure 3 shows the chromatogram obtained for a mixture of triazines the magnetic capillary column applying a magnetic field (a); and without magnetic field (b). As a result of the magnetic field interaction, the analytical response of the analytes increased and the analytes eluted at higher retention times, owing to the higher adsorption of the analytes. It should be noted that unknown compounds eluted at similar retention times than simazine and atrazine, however, they were not considered interferent species since satisfactory chromatographic resolution and quantification could be carried out.

**Figure 4:**
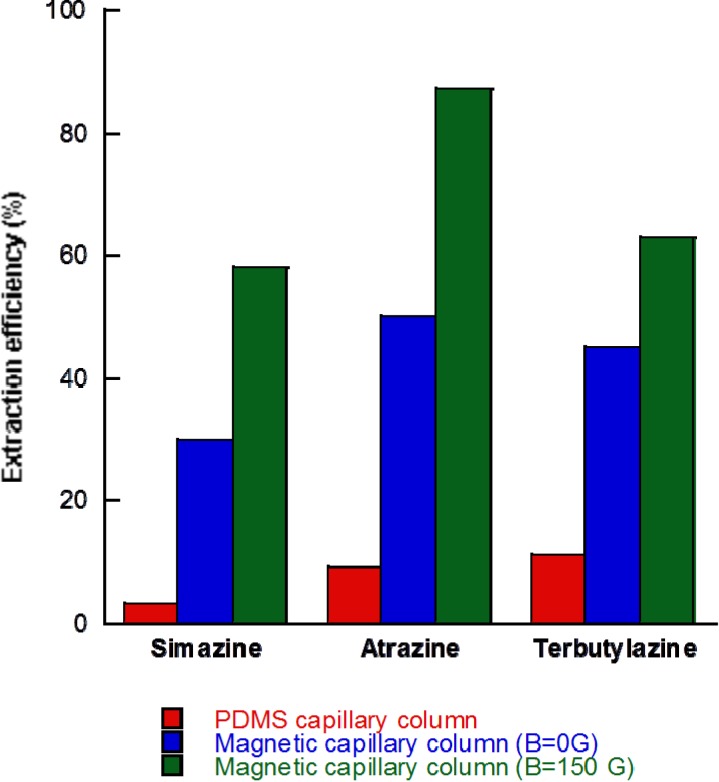
Comparison of the extraction efficiency (%) for simazine, atrazine and terbutylazine with a TRB-5 commercial capillary column, SiO_2_ supported Fe_3_O_4_ capillary column without magnetic field (*B* = 0 G) and with the SiO_2_ supported Fe_3_O_4_ capillary column applying magnetic field (*B*_adsorption_ = 150 G, *B*_desorption_ = 150 G, reverse polarity).

In an attempt to evaluate the benefits of the SiO_2_ supported Fe_3_O_4_ material for triazines, the adsorption of triazines in a typical IT-SPME device using a commercial capillary column (polydimethylsiloxane (PDMS), TRB-5) was studied, under the same conditions (length of the capillary: 15 cm; volume of sample: 100 μL of a mixture of simazine, atrazine and terbutylazine at 30 μg L^−1^). The extraction efficiencies for simazine, atrazine and terbutylazine were 3%, 9% and 11%, respectively. Figure 4 compares the adsorption capacity for the commercial capillary column in IT-SPME modality, the SiO_2_ supported Fe_3_O_4_ capillary column in IT-SPME modality and the SiO_2_ supported Fe_3_O_4_ capillary column in magnetic-IT-SPME modality. Triazines exhibited a higher adsorption on the magnetic capillary column (between 30% and 40%), compared with the typically used commercial capillary column, lower than 15%. It should be noted that these values can be even lower (0.8%–3%) when higher volumes of samples are processed. In addition, the use of the magnetic capillary column in the magnetic-IT-SPME modality even improved these results, and in some cases, such as for atrazine, the extraction efficiency was almost quantitative (87%).

Moreover, the magnetic capillary column also exhibited a good stability for its use in on-line devices. Fe_3_O_4_ NPs were stable inside of the SiO_2_ matrix, since this matrix avoided NPs interaction, agglomerations and also their incorporation to the column flow [19]. In fact, the capillary column was used almost 200 times without loss in the adsorption capacity.

Taking into account the features of this methodology, some analytical characteristics were established in order to characterize the procedure. The analytical response was lineal in the working concentration interval from 3 to 40 μg L^−1^. Table 3 shows the detection limit (LOD, calculated as 3*S*_blank_/*b*, *b*: slope of the calibration graph), quantification limit (LOQ) and precision values (at 2.5 μg L^−1^; expressed as relative standard deviation, RSD) values for simazine, atrazine and terbutylazine, respectively.

**Table 3:** Detection limit (LOD), quantification limit (LOQ) and relative standard deviation (RSD) for simazine, atrazine and terbutylazine achieved with Magnetic-IT-SPME-CapLC-DAD.

Compound	LOD (μg L^−1^)	LOQ (μg L^−1^)	RSD (%)
Simazine	0.4	1.4	10
Atrazine	0.3	1.1	9
Terbutylazine	0.3	1.0	7

These results demonstrated that this methodology has direct applicability for the determination of triazines in environmental samples, such as water samples. The LODs are comparable but a little bit higher than those previously reported in the literature [23]. Note, however, that in this work, we have processed 100 μL of samples; thus, the sensitivity can be even improved by processing higher volumes of water samples.

### 2.3. Application of the Magnetic Capillary Column for the Analysis of Triazines in Water Samples

Magnetic-IT-SPME-CapLC has been proposed for analytical purposes, therefore, in this section we evaluated the applicability of this methodology to analyse real water samples. For this aim, four river water samples were analyzed. The results showed that the simazine, atrazine and terbutylazine were not detected at the concentration levels assayed.

A recovery study was also carried out in order to evaluate the possible matrix effects, caused by components of the water samples. This effect was evaluated by spiking the water samples with a mixture of triazine (2.5 μg L^−1^, each). The recovery values were between 99% ± 1% and 110% ± 5%. Therefore, Magnetic-IT-SPME-Cap-LC did not shown matrix effects under the optimized conditions.

## 3. Experimental Section

### 3.1. Synthesis of Fe_3_O_4_ NPs and SiO_2_ Supported Fe_3_O_4_ Capillary Columns

Syntheses were carried out by the method described in [19]. Fe(acac)_3_ (0.706 g, Aldrich) 1,2-hexanodiol (2.013 g, Aldrich), oleic acid (1.695 g, Aldrich) and oleyamine (1.605 g, Aldrich) were mixed in 20 mL of phenyl ether (Aldrich) under Ar in order to ensure an inert atmosphere. After refluxing the mixture during 30 min at 263 °C, ethanol (80 mL) was added. Then, the mixture was centrifuged and redissolved in 20 mL of hexane. Finally, water soluble NPs Fe_3_O_4_-CTAB were prepared.

The silica supported Fe_3_O_4_ nanomaterial was synthetized using the method described earlier [19], in this procedure PEG (0.9 g) and urea (0.9 g) were dissolved in 10 mL of acetic acid (10 mM). Then, 2.5 mL of this solution were mixed with Fe_3_O_4_-CTAB water dispersion (1 mL) and the pH was adjusted to 11 with NaOH (1 M). After, TEOS was added (1 mL) to the solution and stirred until a homogenous gel was obtained.

Finally, a fused silica capillary column (*id* = 75 μm) was pretreated with NaOH (1 M), and then the gel was injected into the capillary column. Rapidly, the capillary ends were sealed and place into an oven. The capillary coating was achieved using the temperature program described in [19].

### 3.2. Physical Characterization

Magnetic characterization was carried out in a MSMS Squid Magnetometer (Quantum Design, San Diego, CA, USA) with variable temperature (*T* = 2 K).

### 3.3. Instruments and Chromatographic Conditions

The capillary chromatographic system consisted of a liquid chromatography isocratic capillary pump (Jasco Corporation, Tokyo, Japan) connected to a UV-Vis diode array detector 1200 series (Agilent, Waldbronn, Germany) with a 80 nL flow cell.

The separation of the triazines was carried out with a particulate column Zorbax C18 (150 mm × 0.5 mm, 3.5 μm). The mobile phase was a mixture of methanol:water 70:30 at a flow rate of 6 μL min^−1^. All solvent were filtered through 0.45 mm nylon membranes (Teknokroma) before use.

### 3.4. Magnetic—IT-SPME Device

**Figure 5:**
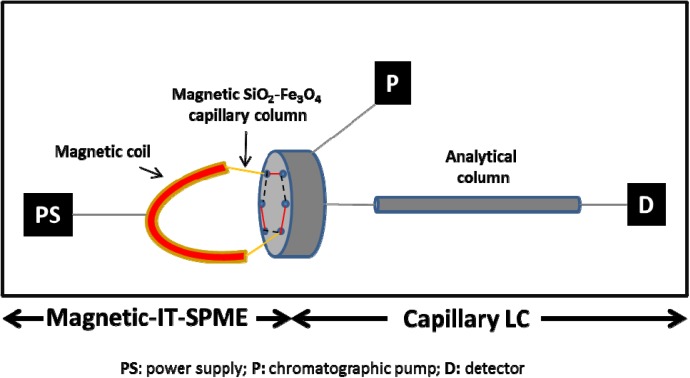
Schematic diagram of the Magnetic-IT-SPME-Cap-LC system. (---) adsorption (load position of the injection valve); and (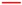) desorption (injection position of the injection valve).

Figure 5 shows the schematic diagram of the Magnetic-IT-SPME coupled to a CapLC system. The SiO_2_ supported capillary column (15 cm) wrapped with a magnetic coil, was connected to the six port injection valve of the CapLC system. The magnetic coil was connected to a power supply (PS) in order to control the magnetic field intensity. The adsorption of the analytes was carried out in the load position of the injection valve (---), 100 μL of samples were manually processed at different magnetic fields (from 50 to 400 G). Then, the analytes were desorbed and transferred to the analytical column for their separation and detection by rotating the valve to the inject position (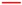) at the same time that the polarity was changed [19]. After each injection, the capillary column was rinsed with 300 μL of methanol.

### 3.5. Computancional Methods

All calculations were carried out with the Gaussian 09 suite of programs [24]. Density functional theory [25,26] calculations (DFT) have been carried out using the B3LYP [27,28] exchange-correlation functionals, together with the standard 6-31G** basis set [29]. The inclusion of solvent effects has been considered by using a relatively simple self-consistent reaction field (SCRF) method [30,31] based on the polarizable continuum model (PCM) of Tomasi’s group [32,33,34]. Geometries have been fully optimized with PCM. The solvent we used was H_2_O (common solvent in HPLC). Calculations of magnetic susceptibility using gauge including atomic orbital method (GIAO) were carried out using NMR = Susceptibility.

### 3.6. Analysis of Water Samples

River water samples collected from several points of the Comunidad Valenciana were analysed. They were filtered through 0.45 μm nylon membranes (Teknokroma) in order to remove any particulate matter, and directly processed in the Magnetic-IT-SPME-CapLC system. The analyses were carried out in triplicate.

## 4. Conclusions

Rapid developments on the synthesis of magnetic nanomaterials have given to the analytical nanotechnology attractive materials to improve several steps of the analytical procedure. Among them, the use of magnetic nanomaterials in the sample pretreatment step is one of most exploited, although, most of these nanomaterials have been used in off-line procedures. Herein, in this work we have demonstrated the applicability of SiO_2_ supported Fe_3_O_4_ magnetic nanomaterial to develop an on-line extraction and preconcentration tool, Magnetic-IT-SPME, for the determination of triazines. The magnetic nanomaterial deposited on a capillary column, takes advantage of the adsorption properties of the sorbent combined with the influence of an external magnetic field on the analytes to enhance the adsorption capacity. Reduction of the analysis time and increment of the extraction efficiency are the most attractive characteristic of this analytical procedure. In addition, the use of this material in an on-line device represents a cost effective analytical methodology for the determination of triazines in environmental samples. This procedure has been successfully applied for determining triazines in the water samples.
